# Few Outflow Problems With a Self-locating Catheter for Peritoneal Dialysis: A Randomized Trial

**DOI:** 10.1097/MD.0000000000002083

**Published:** 2015-12-07

**Authors:** Bernd G. Stegmayr, Wolfgang Sperker, Christina H. Nilsson, Christina Degerman, Sven-Erik Persson, Jan Stenbaek, Conny Arnerlöv

**Affiliations:** From the Departments of Public Health and Clinical Medicine and Surgical and Perioperative Science, Urology and Andrology, Umea University, Umea, Sweden

## Abstract

We developed a technique for direct start of peritoneal dialysis. Using a coiled or straight Tenckhoff catheter often results in obstruction of flow. A self-locating Wolfram catheter is on the market. It is not clarified if this results in a benefit.

The primary aim of this study was to perform a randomized investigation to clarify if the use of a self-locating peritoneal dialysis (PD) catheter would result in different flow problems than a straight Tenckhoff catheter.

A total of 61 insertions were made who were randomized and received either a straight Tenckhoff (n = 32) or a self-locating Wolfram catheter (n = 29).

A previously described operation technique allowed immediate postoperative start of dialysis. Seven straight Tenckhoff catheters had to be changed into self-locating catheters, and none vice versa, due to flow problems (*P* = 0.011). An early leakage resulted in temporarily postponed PD in 4 patients.

This study showed that using the present operation technique the self-locating PD-catheter causes fewer obstruction episodes than a straight Tenckhoff catheter. This facilitates immediate postoperative start of PD.

## INTRODUCTION

It is often difficult to start acute peritoneal dialysis ^1^ because of the need for a break-in period (no peritoneal dialysis) of 14 days postoperatively for most operative techniques used.^2^ The reason to use a delayed start is to avoid leakage and outflow obstruction. Although the use of a swan neck catheter versus a straight outer part of the Tenckhoff catheter does not differ,^3^ the use of a coiled inner part causes more complications than when using a straight inner part.^4–7^ The coiled catheters are more frequently caught by the omentum and dislocated.

To avoid dislocations a “self-locating” catheter was invented.^8^ The catheter contains a tip with a tungsten (Wolfram) weight. This catheter is meant to be self-locating toward the fossa and thereby avoid dislocation. Beneficial data have been shown in several observational studies.^8–11^ The benefit was also shown in an Italian multicenter study.^8^ After detailed explanation and information, patients were invited to choose between the 2 types of catheter. With rare exceptions, the same surgeon in each center implanted the catheters. Insertion techniques could vary between the centers.

As we have a surgical technique using 3 purse string sutures, we start peritoneal dialysis directly postoperatively both in acute and chronic dialysis settings.^12,13^ The technique results in few problems with leakage.^14^ However, obstructive episodes occur intermittently requiring adjustment using either a bended stylet ^15,16^ or reoperation.

The primary aim of the study was to analyze if the Wolfram catheter would perform better than the straight peritoneal dialysis catheter regarding filling and outflow problems when starting peritoneal dialysis.

## MATERIAL AND METHODS

We had previously established the benefit using a straight double cuff Tenckhoff before a coiled catheter.^4^ Thereafter, at our hospital, insertions were performed using either a straight or a self-locating Wolfram catheter. To clarify if there was a benefit using one or the other we decided to perform a systematic quality assessment study using randomization. If failure would occur the other type of catheter should be used. All patients were informed and consented to participate in the setting. All patients started their dialysis program using peritoneal dialysis (PD). The study started February 2007 and ended June 2013. A peritoneal dialysis nurse made randomization from envelopes and provided the surgeon with the respective catheter. Once the patient was accepted for peritoneal dialysis by the physician in charge, there were no exclusion criteria for randomization. The study has attained local ethical committee approval (Ethical Committee, Umea, Sweden, 2012-181-31M at June 5, 2012) and the ClinicalTrials.gov Identifier: NCT02347592.

As numerous patients are unaware of their chronic uremic condition when they are confronted with health care for the first time they need to start dialysis rather acute. Other patients postpone the dialysis start until late by other reasons. If they start acute using hemodialysis few will change to PD later. Starting directly (acute) with PD results in lesser drop out off PD. The terminology acute in this text refers to both acute and chronic kidney disease patients. In this study only chronic patients were included.

A total of 61 insertions were performed either to a straight Tenckhoff (n = 32, 53% men) or a self-locating Wolfram catheter (n = 29, 69% men). The study was performed on a University Hospital. The mean age of the group randomized to straight Tenckhoff did not differ with the group of patients with a self-locating catheter (Table 1). The various diagnoses for chronic kidney disease are given in Table 1.

**TABLE 1:**
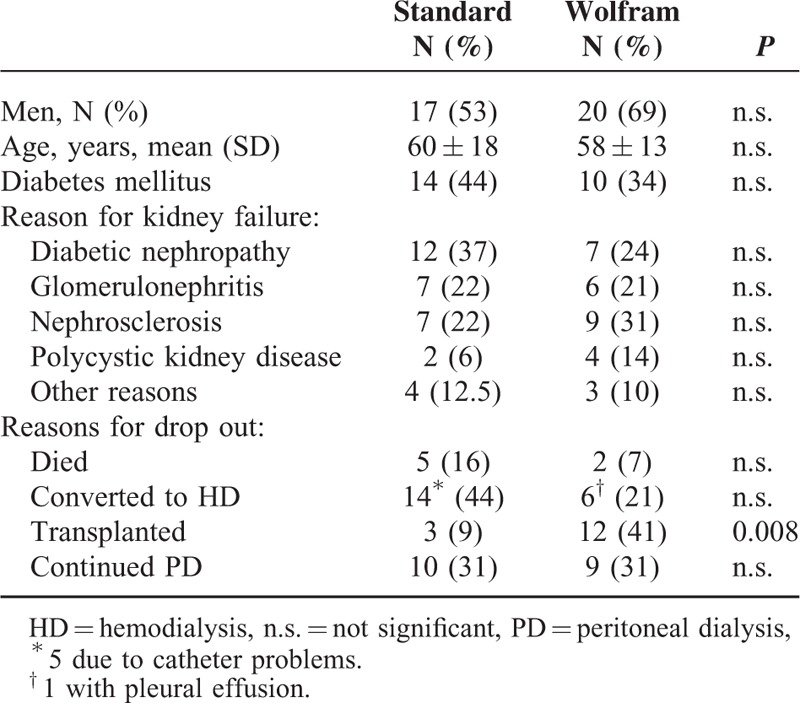
Baseline Data of Patients Who Either Receive a Double Cuff Tenckhoff (Standard, N = 32) or a Self-Locating Wolfram Catheter (Wolfram, N = 29) and Reasons for Ending PD

Catheters used were from Fresenius Medical Care (Homburg, Germany), either a straight double-cuffed Tenckhoff or a double-cuffed Wolfram (tungsten, heavy tip) catheter by Di Paolo.

### Operation Technique (Figure 1 A–H)

**FIGURE 1:**
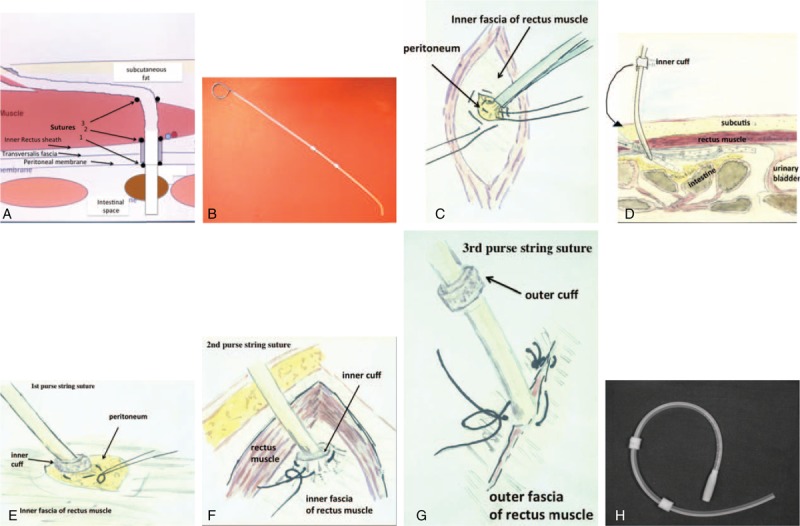
(A) Cross-section at the level of ∼3 cm below the navel showing the layers where the catheter is placed during the surgery. The inner cuff of the catheter is fixed between the peritoneum and the inner fascia by 2 purse string sutures. A third suture closes the outer fascia around the catheter before the catheter proceeds in the subcutaneous tissue toward the exit. (B) A bended stylet is inserted into a Tenckhoff catheter before insertion into the abdomen. This technique helps to guide the catheter toward the left fossa in the pelvis area. (C) The peritoneal membrane is exposed and lifted by a forceps. A purse string suture is fixed before a small incision allows insertion of the catheter. (D) The catheter insertion is guided by the stylet. By a rotation the tip of the catheter is turned toward the front of the peritoneum, thereby avoiding that the catheter is embedded in the intestine. Thereafter the catheter is located into the right position before the stylet is partly withdrawn. (E) The first purse string suture is tightened around the catheter below the inner cuff. (F) The inner cuff is embedded between the peritoneum and inner fascia and the second purse string suture fixes its position and tightens the channel. (G) The catheter exits the outer fascia in a direction upwards and to the right before bending into the subcutaneous space. The exit through the fascia is closed around the catheter to fix and tighten the position. (H) A self-locating catheter can be inserted in the same way.

A few experienced surgeons, specialized in general and vascular surgery, performed catheter insertions. A strict protocol for the operative technique was used. The insertion technique is the same for both types of double cuffed peritoneal dialysis catheters, whereas the difference is at the inner end of the Wolfram catheter, holding a metal weight. The insertion of the catheter is performed in the operation theatre in local anesthesia, and often mild sedation of the patient. Incision of the skin and the right anterior rectus sheath is performed. The fibers of the rectus abdominal muscle are separated and a small hole is made in the posterior rectus sheath. Thereafter, the peritoneal membrane is identified and a small incision of the membrane allows the catheter to be located into the left lower fossa. The location of the catheter is facilitated using a stiff slightly bended stylet with a blunt end. The stylet is placed within the catheter ^16^ and withdrawn when the catheter is in the right location.^12,13^ The inner cuff is placed outside the peritoneum and beneath the posterior rectus sheath and angulated toward the left lower fossa with 2 purse string sutures. The first suture tightens the membranes around the catheter and the second suture further tightens the membranes around the cuff causing a watertight seal. The outer fascia is closed around the catheter with the third row of sutures. Finally, a tunnel is created toward the right side of the abdomen where the exit site is placed and the outer cuff located 2 cm inside of the exit through the skin. Using the 3-purse-string-suture technique the catheter is tightened and fixed by the tissue around the catheter. This does restrict movement of cuff and catheter within the channel created. The location of the catheter between the peritoneal opening and the inner fascia is in direction toward the left lower abdomen and the curve of the subcutaneous tunnel is smooth to avoid kinking of the catheter. During the operation the location of the catheter tip in the left lower fossa is usually verified by x-ray. In addition filling and emptying the abdomen before placing the final sutures tested the function of the catheter (see also Appendix). Based on a randomized study, in the first postoperative dialysis bag, instilled directly after return from operation, cefuroxime (250 mg/L) is used as antibiotic prophylaxis.^17^ The patient is kept in a supine position for 2 h, before allowed to walk around. When the patient is out of bed with PD-fluid in the abdominal cavity a surgical girdle is used during the first 2 to 4 postoperative days to avoid distension of the wound Figure 1.^18^

Primary outcome measures: problems related to the type of catheter were considered to be filling and outflow dysfunction.

Outflow dysfunction was defined as inability to repeatedly drain >500 mL PD fluid within 40 min. An x-ray investigation of the location of the catheter was performed to decide if reoperation should be performed or not.

Secondary outcome measure: early and late leakages were considered as problems related to the operation technique in the tissue area (not enabling tightness between abdominal cavity and exit).

When outflow failure occurred, repeated attempts were made to overcome the problem. Initially the patient was informed to change into various positions, using laxatives. When dysfunction remained, x-ray was performed. If omental wrapping was not obvious attempts were made using fibrinolytic agents such as heparin and even attempts were made using correction with a bended stylet. Thereby the stylet is inserted into the catheter to try to correct the position of the catheter without surgery.^16^

If total obstruction was present or if outflow or inflow dysfunction remained and surgery was decided this was defined as outflow failure (death of catheter in the survival analysis).

Early and late leakages were used as the secondary outcome variable as leakage is 1 important reason for interruption of the dialysis process. As the self-locating catheter has a wider tip it needs a larger entry hole through the peritoneum than a regular catheter. This larger hole may increase the risk for early leakage.

### Statistics

Sample size was estimated by assuming at least 30% differences between groups. Interim results were obvious due to an open study design. Clinical differences urged for an interim analysis after June 2013. Fisher's test was used for the comparison of catheter patency. For mean value calculations, the Student *t* test was used. Kaplan–Meier analyses were performed. A 2-tailed *P* value of < 0.05 was considered statistically significant. IBM SPSS software version 22 was used. Kaplan–Meier analyses were performed with R on an Apple MacBook Pro, System 10.10.1.

R Core Team (2014; R: A language and environment for statistical computing. R Foundation for Statistical Computing, Vienna, Austria. URL http://www.R-project.org/).

## RESULTS

Start of peritoneal dialysis was initiated directly postoperatively in all 61 insertions (100%). Median follow-up was 10 months (range 1–76, mean 15 ± 17 months).

### Primary Outcome Measure

Obstructions resulted in reoperation in 7 of 32 inserted straight Tenckhoff catheters and none of 29 self-locating Wolfram catheter insertions (Fisher's test, 2-tailed, *P* = 0.01). These 7 straight Tenckhoff catheters were changed into self-locating catheters, and none vice versa (*P* = 0.011).

**FIGURE 2:**
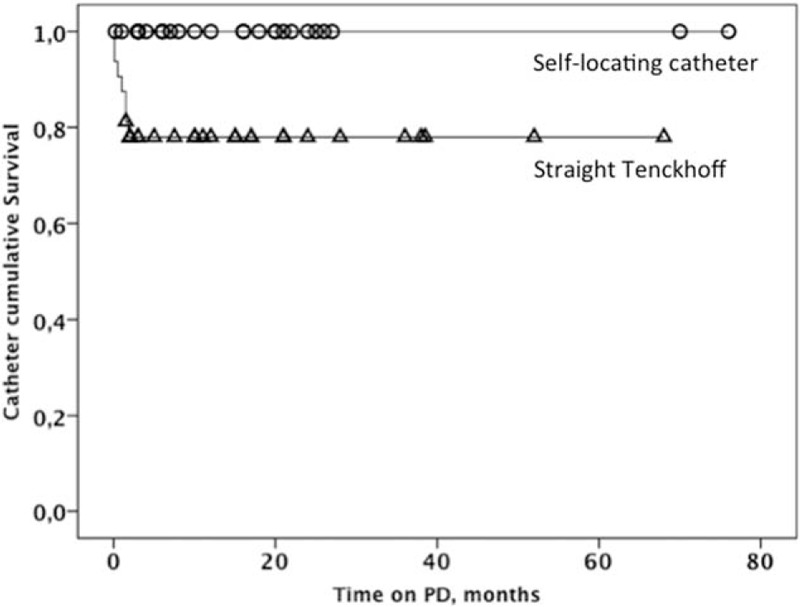
Kaplan–Meier distribution of catheter survival in relation to need of reoperation due to outflow failure. The self-locating Wolfram catheter is shown with open circles and the straight Tenckhoff catheter with open triangles.

The survival curve of the catheters showed a worse outcome for the straight Tenckhoff (Breslow analysis, *P* = 0.01, Fig. 2), all problems happening during the early phase of PD. The flow problems arose within the first 2 months. If a straight Tenckhoff catheter was patent at that time there was no difference in patency between catheters. The catheters were thereafter functioning well with both techniques (Fig. 2). The reasons for patients to end the PD program are given in Table 1.

### Secondary Outcome Measure

Leakage was present in 4 (6.5%) of the placements during the first days (1of those with self-locating catheter and 3 with the straight Tenckhoff). The patients were kept off PD (dry) for 6, 8, 14, and 17 days, respectively. Restart was without leakage. In general, their tissue was considered very soft by the surgeon. No late leakage developed.

A total of 7 surgeons had inserted catheters through the period. Three had inserted most (≥5 insertions/surgeon) whereas 4 were less experienced. The extent of outflow problems that developed postoperatively could not be related to their number of insertions (Fisher's test).

No unintended harm occurred in any of the patients and groups.

## DISCUSSION

This study shows that the use of a self-locating catheter is superior to the straight catheter regarding in and outflow problems. Notable is that our insertion technique is meant to allow direct use of the PD catheter without any break-in period. This may differ from data with those who have long break-in periods.

In the multicenter study by di Paolo et al,^8^ patients were invited to choose between the 2 types of catheter. As 78% selected the self-locating catheter there might be a selection bias or an eventual influence by staff. The differences in obstruction in favor to the self-locating catheter were significant but not as extensive as in this study (9 versus 7 %) versus >20% and 0% in the present study. Although that study does not report of surgical insertion techniques and break-in periods used both studies indicate the benefit of a self-locating catheter. In the present study the combination of using a prompt start of PD with the described insertion technique and the self-locating catheter may favor that more patients will accept to start on peritoneal dialysis. In out-program many patients are on the waiting list for transplantation. As they are active in daily life maintaining their work a proper location of the catheter is essential to maintain in the PD program. We believe that the main reason for obstruction of a PD catheter is due to a dislocation of the tip of the catheter caused for example by a patient that is in motion, bending forward and dislocating the catheter upward or an omentum catching and obstructing a moving catheter tip. By using the self-locating Wolfram catheter the weight at the tip keeps the catheter in a position in the pelvis of the patient. This will protect from dislocation when the patient is moving. However, some patients may complain of a discomfort in the pelvis, probably caused by the pressure of the weight or a too long catheter. We suggest that the length is carefully measured preoperatively. If the catheter is too short, that is, in an obese patient, the tip may be fixed within the omentum and result in drainage failure. To facilitate the insertion of the catheter, we consequently use a curved stylet ^16^ inserted into the catheter during placement. This helps to direct the tip of the catheter into its right position, and routinely the position of the catheter is verified by x-ray preoperatively. This curved stylet can also be used to try to reposition a misplaced catheter.

When obstruction occurs, other measures than described in this study may also be tried if experience exists such as using single-port laparoscopy for salvaging outflow failure from omentum wrapping.^19^

A limitation of the study was that the surgeon was not blinded to the catheters inserted. It is important the tip of the self-locating catheter is located right at start to avoid discomfort and invagination into the omentum. This is facilitated using the bended stylet described and a follow-up x-ray check preoperatively. The larger hole created in the peritoneum for insertion of the metal tip may increase the risk for early leakage although the operation technique was kept the same. If dislocation occurs it is trickier to adjust the position of the self-locating catheter with a stylet due to the weight at the tip.

In conclusion, this study showed that using the present operation technique the self-locating PD-catheter causes fewer obstruction episodes than a straight Tenckhoff catheter. This facilitates immediate postoperative start of PD.
